# ABLIM1: A novel oncogenic E3 ligase in colorectal cancer

**DOI:** 10.3389/fonc.2025.1718420

**Published:** 2025-11-20

**Authors:** Ganesh Kumar Barik, Osheen Sahay, Sehbanul Islam

**Affiliations:** 1 Cancer Biology Division, National Centre for Cell Science, Savitribai Phule Pune University, Pune, Maharashtra, India; 2 Department of Cancer Biology, Abramson Family Cancer Research Institute, Perelman School of Medicine, University of Pennsylvania, Philadelphia, PA, United States

**Keywords:** E3 ubiquitin ligase, ABLIM1, colorectal cancer, LIM proteins, ubiquitination, signaling pathway

The Lin-11, Isl-1 and Mec-3 (LIM) domain, discovered in the late 1980s, is an evolutionarily conserved, cysteine- and histidine-rich, Zinc finger domain that mediates protein-protein interactions. LIM domain-containing proteins constitute a large family of nuclear and cytosolic proteins (70 members in humans) that are divided into 4 groups based on domain composition and arrangement, and the subcellular localization (1). The LIM proteins display diverse cellular functions, including cytoskeleton organization, gene regulation, and cell fate determination. Since LIM proteins have one or more Zn-finger motifs like RING, they are proposed to have E3 ligase activity (2, 3). Indeed, Tanaka et al. identified STAT-interacting LIM protein (SLIM), also called PDLIM2/Mystique, as the first nuclear LIM domain-containing E3 ligase that targets STAT proteins and p65 for ubiquitination-mediated proteasomal degradation and thereby inactivates the STAT and NF-κB signaling (4–6). These findings suggest that LIM proteins might constitute a new family of E3 ubiquitin ligases; however, only a handful have been verified to have E3 ligase activity. Furthermore, the investigation into the role of LIM proteins as E3 ligases within the realm of cancer remains underexplored.

Actin-binding LIM protein 1 (ABLIM1), a member of the ABLIM family of group-3 LIM proteins, consists of 4 LIM domains at the N-terminal, and a coiled-coil and HP domain at the C-terminal region. ABLIM1 is initially characterized as a cytoskeleton protein interacting with the actin filaments and regulating actin networks. A few studies reveal low levels and a tumor suppressor role of ABLIM1 in melanoma, nasopharyngeal carcinoma, and glioblastoma; however, the underlying mechanisms governing ABLIM-mediated tumorigenesis remain elusive.

In a recent study published in *Cell Death and Differentiation*, He et al. unravelled an unknown role of ABLIM1 in CRC, shedding light on its distinctive functions and elucidating the intricate molecular mechanisms at play (7). The authors showed that elevated ABLIM1 expression in CRC patients correlated with shorter disease-free survival, suggesting its potential as a prognostic biomarker for predicting disease relapse in CRC patients. Furthermore, using *in vitro* and *in vivo* studies, the authors showed that ABLIM1 functions as an oncogene to promote the growth and liver metastasis of CRC cells, unlike its previously reported tumor suppressive role in other cancer models. An interesting and novel finding of this study is that ABLIM1 functions as an E3 ubiquitin ligase, which catalyses the ubiquitination and degradation of IĸBα. Since IĸBα is a well-known inhibitor of the NF-ĸB signaling pathway, ABLM1-mediated degradation of IĸBα leads to the translocation of NF-ĸB complex (p65/p50) to the nucleus, transcriptional activation of oncogene CCL20. Overall, the study reveals that ABLIM1 functions as an E3 ligase to promote growth and liver metastasis in CRC by activating the oncogenic NF-ĸB-CCL20 axis. This finding underscores the potential of targeting ABLIM1-NF-ĸB-CCL20 oncogenic axis to prevent CRC progression and metastasis (**Figure 1**).

**Figure 1:**
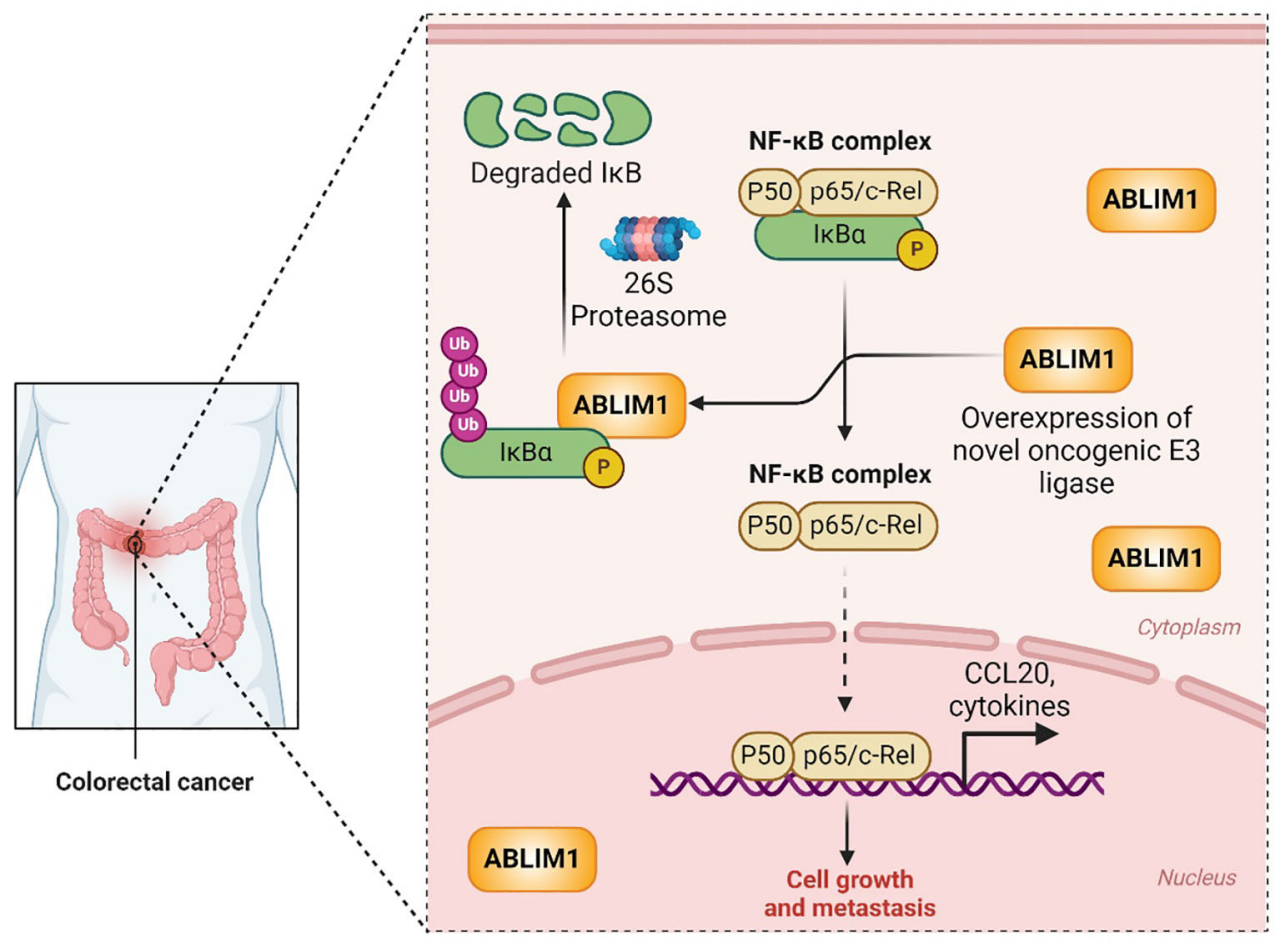
ABLIM1 functions as a novel E3 ubiquitin ligase to drive CRC growth and metastasis via ubiquitination-mediated degradation of IĸBα and activation of NF-ĸB-CCL20 oncogenic signaling. The cartoon on the left shows a patient suffering from CRC. A colorectal cancer cell is zoomed in the right to depict ABLIM1 overexpression and the underlying molecular mechanisms that drive growth and metastasis in CRC. IĸBα sequesters the NF-ĸB complex (P50-P65/c-Rel) in the cytoplasm and thereby inactivates the NF-ĸB signaling. Overexpressed ABLIM1 interacts with and polyubiquitinates IĸBα, leading to its degradation by the 26S proteasome. Thus, the freed NF-ĸB complex enters into the nucleus and transcriptionally activates the expression of CCL20 and other cytokines which ultimately leads to CRC growth and metastasis.

This study holds significant importance from both a basic and clinical perspective. This study discovers another LIM protein, ABLIM1, functioning as an E3 ligase and strengthens the existing hypothesis that the LIM protein family might constitute yet another E3 ligase family. An intriguing revelation emerges as the research unveils that the C-terminal region exhibits autoubiquitination *in vitro*, suggesting that the E3 ligase activity is associated with the HP domain. However, deletion of LIM domains significantly hinders ABLIM1-mediated IĸBα polyubiquitination, indicating that the LIM domain is essential for ABLIM1 catalytic activity. Other studies show similar observations that deletion of LIM domains of LIM proteins like PDLIM7 and Hic-5 significantly decreased their E3 ligase activity (8, 9). Further investigation is crucial to unravel the molecular mechanisms of ABLIM1 catalytic activity, where the C-terminal region houses the E3 ligase activity, while the N-terminal LIM domains play an essential role in maintaining catalytic function. Understanding how these regions cooperate and influence each other at the molecular level will provide valuable insights into the regulation of E3 ligases. In particular, the role of the ABLIM1 C-terminal coiled-coil domain warrants detailed exploration. In TRIM family E3 ligases, coiled-coil domains promote oligomerization that stabilizes the active E3 conformation and properly positions the E2~ubiquitin conjugate for efficient ubiquitin transfer to substrates. It is plausible that ABLIM1’s coiled-coil domain performs a similar function, being critical for its E3 ligase activity and substrate ubiquitination (10). Furthermore, the LIM domains might facilitate a favourable three-dimensional conformation of ABLIM1, facilitating its E3 ligase activity. High-resolution structural studies can shed light on the conformational changes in ABLIM1 induced by the presence or absence of LIM domains. Determining the 3D structures of both the full-length ABLIM1 and its truncated forms can provide visual clues on how these domains contribute to the protein’s overall architecture and function. Studies have also shown that PDLIM2 E3 ligase, along with PDLIM7 LIM E3 ligase or MKRN2 RING E3 ligase, synergistically promotes polyubiquitination and proteasomal degradation of p65 (8, 11). This suggests that the LIM domain might interact with other proteins, enhancing the overall E3 ligase activity of LIM proteins. Therefore, unravelling the specific interactomes of LIM proteins by proteomic analysis will be instrumental in delineating their E3 ligase activity. Notably, the LIM domain of PDLIM2 can catalyse polyubiquitination of STAT3 *in vitro*, suggesting that the LIM domain might have catalytic activity (6). Therefore, further in-depth study is required to investigate which LIM proteins have E3 ligase activity.

The study is also significant from a clinical point of view. As E3 ligases are emerging as potential therapeutic targets in various diseases, understanding the role of ABLIM1 and other LIM proteins in ubiquitination processes may pave the way for developing targeted interventions. Modulating the activity of these proteins could offer novel strategies for therapeutic interventions in conditions where ubiquitin-mediated processes are implicated. Since ABLIM1 is upregulated in CRC, inhibiting its expression through siRNAs might robustly reduce tumor growth and metastatic burden in CRC patients. Structure-based drug design or High-Throughput Screening (HTS) using compound libraries could be employed to identify novel small molecule inhibitors or molecular glues that modulate their activity or degradation. In addition, PROTACs could be designed to recruit an E3 ligase such as CRBN or VHL to ABLIM1, marking it for degradation and effectively reducing its cellular levels. Furthermore, ABLIM1 is the upstream regulator of the oncogenic NF-ĸB signaling pathway, and NF-ĸB small molecule inhibitors are approved or in clinical trials. Combination therapy targeting both ABLIM1 and the NF-ĸB pathway might give better anti-cancer therapeutic options.

The findings from this study pose a series of interesting and exciting questions. The first question revolves around the dual nature of ABLIM1 in cancer- how does this protein serve both tumor-suppressive and promoting functions, and what molecular mechanisms underlie this duality? In CRC, activation of the Wnt/β-catenin pathway and mutations in KRAS and TP53 may elevate the basal NF-κB signaling tone, thereby amplifying the effect of ABLIM1-mediated IκBα degradation (12, 13). It is possible that ABLIM1 binds different substrates or partners in different cancer types. Cancers like melanoma, nasopharyngeal carcinoma, and glioblastoma may lack specific cofactors required for IκBα ubiquitination, limiting ABLIM1’s E3 ligase function. In contrast, in CRC, the availability of such cofactors may redirect ABLIM1 activity toward NF-κB activation and tumor progression. Moreover, upstream kinases such as IKK might be overexpressed in CRC, phosphorylate IκBα, and promote ABLIM1 activity in a phosphorylation-dependent manner. Further, investigating the mutational status of ABLIM1 in CRC could help to identify genetic alterations that underlie its cancer-specific regulatory mechanisms. What is the correlation between ABLIM1, NF-ĸB, and CCL20 in CRC patients? The observed ubiquitination of NF-ĸB pathway members by various LIM E3 ligases, including PDLIM1, PDLIM2, PDLIM7, and ABLIM1, raises the intriguing question of functional conservation. PDLIM1 inhibits NF-κB–mediated inflammatory signaling by sequestering the p65 subunit of NF-κB in the cytoplasm (14). PDLIM2, a nuclear LIM E3 ligase, sequesters and promotes p65 degradation to restrain NF-κB signaling induced by TLR stimulation (5). In addition, HSP70 (heat shock protein 70) facilitates PDLIM2-mediated p65 degradation, thereby inhibiting NF-κB signaling (15). PDLIM7, in cooperation with PDLIM2 and p62/SQSTM1, also promotes p65 turnover to attenuate inflammatory responses (8). ABLIM1 expands this repertoire by targeting IκBα rather than p65, highlighting node specificity within the NF-κB pathway (IκBα vs. p65). A comprehensive understanding of whether these LIM E3 ligases share common regulatory mechanisms or exhibit unique roles within the NF-ĸB signaling pathway is essential for deciphering their collective impact. Furthermore, unravelling the stimulus or signaling pathways that regulate ABLIM1-mediated IĸBα degradation is crucial for understanding its precise role in the NF-ĸB pathway. It remains to be explored whether this regulation is phosphorylation-dependent and whether kinases, particularly IKKs, play a role in the regulation of ABLIM1-mediated NF-ĸB regulation. Further, whether and how ABLIM1 regulates the ubiquitination of other NF-ĸB members and thereby regulation of non-canonical NF-ĸB signaling pathway remains elusive. Beyond ABLIM1, an investigation into the E3 ligase activity of other ABLIM proteins (ABLIM2, ABLIM3, ABLIM4) is warranted to uncover potential functional conservation or divergence within the ABLIM family. The investigation can extend to LIM-domain-only (LMO) proteins, known for pivotal roles in cancer (16). ABLIM1, just like most of the LIM proteins, is localized in both the cytoplasm and the nucleus, whereas some are confined to the nucleus only. It would be interesting to explore how the localization affects the E3 ligase activity of these LIM proteins and what their substrate proteins are. Despite lacking additional functional domains, could LMO proteins possess E3 ligase activity? Unravelling their potential contribution to cellular processes and their involvement in cancer biology could present novel insights into the regulatory landscape of LIM proteins. Addressing these questions will enhance our understanding of LIM proteins in general and ABLIM1 in particular in normal physiology and cancer biology, providing valuable insights for future research and guiding precision medicine approaches for cancer treatment that ultimately improve patient outcomes.

## References

[1] Sala S Ampe C . An emerging link between LIM domain proteins and nuclear receptors. Cell Mol Life Sci. (2018) 75:1959–71. doi: 10.1007/s00018-018-2774-3, PMID: 29428964 PMC11105726

[2] Capili AD Schultz DC Rauscher FJ III Borden KL Solution structure of the PHD domain from the KAP-1 corepressor: structural determinants for PHD, RING and LIM zinc-binding domains. EMBO J. (2001) 20:165–77. doi: 10.1093/emboj/20.1.165, PMID: 11226167 PMC140198

[3] Matthews JM Bhati M Lehtomaki E Mansfield RE Cubeddu L Mackay JP It takes two to tango: the structure and function of LIM, RING, PHD and MYND domains. Curr Pharm Des. (2009) 15:3681–96. doi: 10.2174/138161209789271861, PMID: 19925420

[4] Tanaka T Soriano MA Grusby MJ SLIM is a nuclear ubiquitin E3 ligase that negatively regulates STAT signaling. Immunity. (2005) 22:729–36. doi: 10.1016/j.immuni.2005.04.008, PMID: 15963787

[5] Tanaka T Grusby MJ Kaisho T PDLIM2-mediated termination of transcription factor NF-kappaB activation by intranuclear sequestration and degradation of the p65 subunit. Nat Immunol. (2007) 8:584–91. doi: 10.1038/ni1464, PMID: 17468759

[6] Tanaka T Yamamoto Y Muromoto R Ikeda O Sekine Y Grusby MJ . PDLIM2 inhibits T helper 17 cell development and granulomatous inflammation through degradation of STAT3. Sci Signal. (2011) 4:ra85. doi: 10.1126/scisignal.2001637, PMID: 22155789

[7] He Y Shi Q Ling Y Guo H Fei Y Wu R . ABLIM1, a novel ubiquitin E3 ligase, promotes growth and metastasis of colorectal cancer through targeting IĸBα ubiquitination and activating NF-ĸB signaling. Cell Death Differ. (2024) 31:203–216. doi: 10.1038/s41418-024-01256-y, PMID: 38228802 PMC10850134

[8] Jodo A Shibazaki A Onuma A Kaisho T Tanaka T PDLIM7 Synergizes With PDLIM2 and p62/Sqstm1 to Inhibit Inflammatory Signaling by Promoting Degradation of the p65 Subunit of NF-κB. Front Immunol. (2020) 11:1559. doi: 10.3389/fimmu.2020.01559, PMID: 32849529 PMC7417631

[9] Ryan PE Kales SC Yadavalli R Nau MM Zhang H Lipkowitz S Cbl-c ubiquitin ligase activity is increased via the interaction of its RING finger domain with a LIM domain of the paxillin homolog, Hic 5. PloS One. (2012) 7:e49428. doi: 10.1371/journal.pone.0049428, PMID: 23145173 PMC3492284

[10] Lou X Ma B Zhuang Y Xiao X Minze LJ Xing J . TRIM56 coiled-coil domain structure provides insights into its E3 ligase functions. Comput Struct Biotechnol J. (2003) 21:2801–8. doi: 10.1016/j.csbj.2023.04.022, PMID: 37168870 PMC10165346

[11] Shin C Ito Y Ichikawa S Tokunaga M Sakata-Sogawa K Tanaka T MKRN2 is a novel ubiquitin E3 ligase for the p65 subunit of NF-κB and negatively regulates inflammatory responses. Sci Rep. (2017) 7:46097. doi: 10.1038/srep46097, PMID: 28378844 PMC5380948

[12] Cong B Stamou E Pennel K Mckenzie M Matly A Gopinath S . WNT signalling promotes NF-κB activation and drug resistance in KRAS-mutant colorectal cancer. bioRxiv. (2023) 23:572810. doi: 10.1101/2023.12.21.572810, PMID: 41188521 PMC12678608

[13] Cooks T Pateras IS Tarcic O Solomon H Schetter AJ Wilder S . Mutant p53 prolongs NF-κB activation and promotes chronic inflammation and inflammation-associated colorectal cancer. Cancer Cell. (2013) 23:634–46. doi: 10.1016/j.ccr.2013.03.022, PMID: 23680148 PMC3657134

[14] Ono R Kaisho T Tanaka T PDLIM1 inhibits NF-κB-mediated inflammatory signaling by sequestering the p65 subunit of NF-κB in the cytoplasm. Sci Rep. (2016) 5:18327. doi: 10.1038/srep18327, PMID: 26679095 PMC4683373

[15] Tanaka T Shibazaki A Ono R Kaisho T HSP70 mediates degradation of the p65 subunit of nuclear factor κB to inhibit inflammatory signaling. Sci Signal. (2014) 7:ra119. doi: 10.1126/scisignal.2005533, PMID: 25515536

[16] Matthews JM Lester K Joseph S Curtis DJ LIM-domain-only proteins in cancer. Nat Rev Cancer. (2013) 13:111–22. doi: 10.1038/nrc3418, PMID: 23303138

