# Correction: ABLIM1: a novel oncogenic E3 ligase in colorectal cancer

**DOI:** 10.3389/fonc.2025.1753402

**Published:** 2025-12-02

**Authors:** Frontiers Production Office

**Affiliations:** Frontiers Media SA, Lausanne, Switzerland

**Keywords:** E3 ubiquitin ligase, ABLIM1, colorectal cancer, LIM proteins, ubiquitination, signaling pathway

The article type was erroneously given as General Commentary. The correct article type is Opinion.

The original version of this article has been updated.

